# Enhancing Cybersecurity in Distributed Microgrids: A Review of Communication Protocols and Standards

**DOI:** 10.3390/s24030854

**Published:** 2024-01-28

**Authors:** Eyuel Debebe Ayele, Javier Ferreira Gonzalez, Wouter B. Teeuw

**Affiliations:** Academy of Creative Technology, Saxion University of Applied Sciences, M. H. Tromplaan 28, 7513 AB Enschede, The Netherlands; j.ferreiragonzalez@saxion.nl (J.F.G.); w.b.teeuw@saxion.nl (W.B.T.)

**Keywords:** microgrid communication, DER, flywheel technology, data distribution service (DDS), real-time control systems, smart grid protocols, sustainable energy solutions, quality of service (QoS), IEC 61850, Modbus, IDS

## Abstract

The effective operation of distributed energy sources relies significantly on the communication systems employed in microgrids. This article explores the fundamental communication requirements, structures, and protocols necessary to establish a secure connection in microgrids. This article examines the present difficulties facing, and progress in, smart microgrid communication technologies, including wired and wireless networks. Furthermore, it evaluates the incorporation of diverse security methods. This article showcases a case study that illustrates the implementation of a distributed cyber-security communication system in a microgrid setting. The study concludes by emphasizing the ongoing research endeavors and suggesting potential future research paths in the field of microgrid communications.

## 1. Introduction

**Figure 1:**
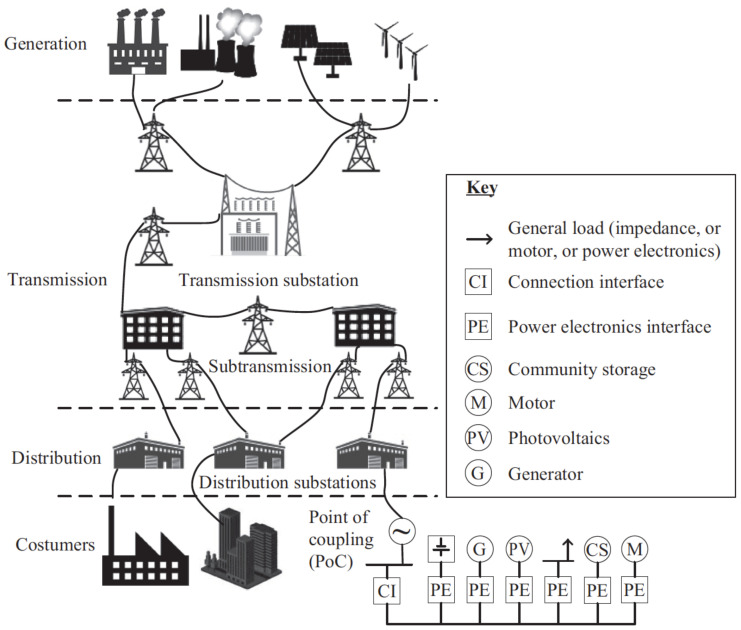
Schematic of a microgrid connected to a distribution network [1].

A microgrid is a comprehensive system that includes energy storage, different energy sources, and loads within a certain boundary. It functions seamlessly, whether it is linked to, or works independently from, the main electrical grid, ensuring a consistent power supply [1,2,3]. Microgrids consist of distributed energy resources (DER) and loads, which may be located in one place or spread throughout an electrical distribution network [1,2]. Their energy source is dependable and can efficiently regulate power distribution in interconnected grid situations or operate autonomously in island mode. Figure 1 illustrates the typical configuration of a microgrid, showcasing its fundamental elements and linkages to large-scale generation, transmission, and distribution networks.

Microgrids must be able to adjust to different operational contexts, such as being connected to the main power grid, operating autonomously or transitioning between these two states. Multiple microgrids can be integrated into the main power grid, with each acting as a key component of the distribution communication network [1,4]. In island mode, a microgrid works independently, providing electricity only to its internal power requirements. The microgrid’s management system interacts with market signals and optimizes features such as peak shaving and frequency regulation. The control system must effectively manage the allocation and utilization of resources, including power production and storage, load management, and ensuring self-sufficient operation [5,6,7].

The illustration in Figure 1 displays a typical microgrid configuration, which includes energy storage, renewable sources such as wind and solar, a microturbine, and various electrical needs. Renewable distributed energy resources (DERs), such as wind and solar power, offer considerable advantages by replacing fossil fuel energy and reducing pollutants. These systems strive to reduce energy losses across transmission, storage, and secondary electrical networks [1,4]. An automated controller is essential for the efficient operation of the intelligent microgrid, as it guarantees the maintenance of optimal frequency and voltage levels. During island mode, the system’s control and management must effectively synchronize its components. The influence of communication technologies in microgrids is contingent on the arrangement of the network and the particular components involved. Uninterrupted and reliable data transmission is essential for the purpose of real-time monitoring [8,9].

**Figure 2:**
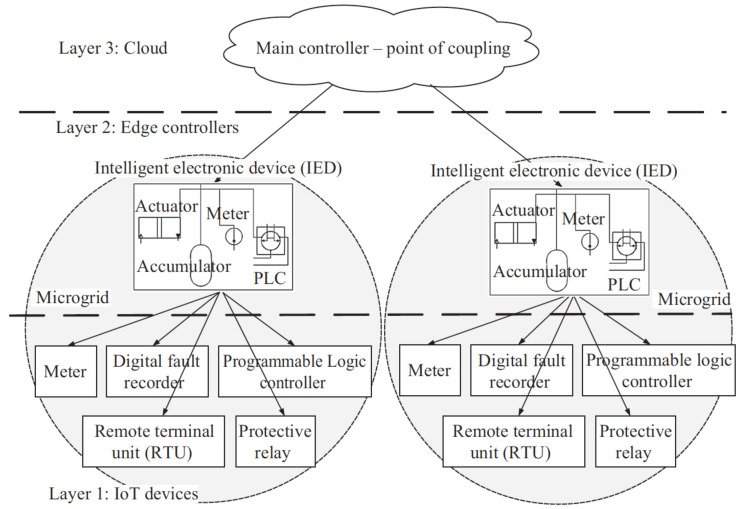
Tri-level communication structure used in microgrid systems [10,13].

The hierarchical structure of microgrid communication architectures typically consists of three tiers (Figure 2) [4,10]. At the top is a central controller that oversees multiple microgrids and the wider smart grid (SG). Energy storage systems (ES) are included to balance loads and enable a smooth transition to islanded operation. Power electronics converters are included to provide improved control and rapid response, allowing for the regulation of varying power consumption by non-essential loads. A local controller (LC) is responsible for managing the state variables of a microgrid, such as phase currents and voltages at the point of common coupling (PCC) [10]. In fact, multiple microgrids can be connected to a single PCC, allowing energy transfer to and from the primary electrical network.

The bottom layer of the microgrid design comprises measuring equipment such as PLCs and safety switches, which regularly track the data flow. The intermediate layer comprises gateway control devices, such as IEDs, whose functions are accountable for immediate processing of information and provisional data retention. The uppermost tier comprises an in-the-cloud platform specifically designed for long-term data storage and performing comprehensive quantitative operations [11,12]. Figure 2 illustrates the variations in computational energy and connectivity demands across each of the three tiers. This requires the implementation of distinct communication methods for every tier. Specifically, the first two layers use both distributed and centralized versions of the home area network (HAN) [10,13] and the local area network (LAN) [13]. The higher levels, in contrast, utilize the neighborhood area network (NAN) [14] and the wide area network (WAN) [14].

Microgrids are a significant development in the distribution of energy. They are defined by their incorporation of energy storage, various energy sources, and loads within specific bounds. Whether operating independently or in tandem with the primary electrical grid, these systems guarantee a reliable power source and demonstrate durability in diverse operational situations [15,16,17]. Nevertheless, despite their increasing ubiquity, there are still significant deficiencies in comprehending and executing efficient cybersecurity measures in these systems, namely in the domain of control communication protocols and standards.

This research aims to fill these research gaps by performing a thorough examination of the available literature and comparing it to the present condition of microgrid cybersecurity. We examine the intricacies of distributed energy resources (DER) in microgrids, investigating their setup and the crucial significance of communication technologies for instantaneous monitoring and control [18,19,20]. The incorporation of sustainable energy sources, such as wind and solar power, into microgrids poses distinct problems and possibilities in terms of upholding cybersecurity. This requires a strong and flexible communication system that can handle both networked and isolated operational modes.

This review paper distinguishes itself from prior reviews by specifically examining the integration and consequences of sophisticated communication protocols, such as the data distribution service (DDS), across different sectors [21,22,23]. We analyze previous case studies that utilize DDS and other protocols, evaluating their effectiveness and suitability for microgrid systems. Moreover, the article emphasizes the existing deficiencies in microgrid cybersecurity, offering valuable perspectives on prospective future developments and breakthroughs in this domain.

The organization of this article is as follows: Section 2 and Section 3 give an overview of the communication infrastructure and its current implementations. Section 4 looks at prior case studies that have employed a distributed data distribution service. Section 5 explains in detail how the prototype for the microgrid testbed system was implemented. Section 7 provides a comprehensive summary of the study, pinpoints potential areas for further research, and addresses related issues.

## 2. Cybersecure Communication in Microgrid

### 2.1. Cybersecurity Overview

**Figure 3:**
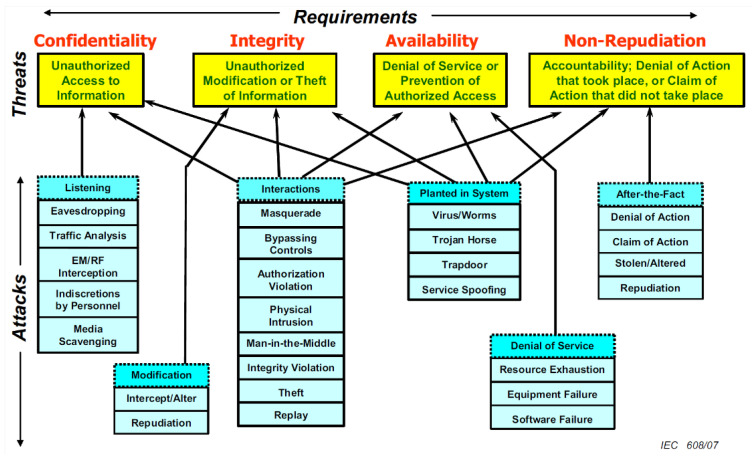
Overview of microgrid security [24].

The IEC 62351 standard [24] lays out the security framework for managing grid systems and sharing information about them. Figure 3 shows an overview of this framework. It demonstrates the link between different cyber security risks and the security requirements necessary to reduce them. These risks are divided into four categories: unauthorized access to data (confidentiality), unauthorized alteration or theft of data (integrity), and non-repudiation, which guarantees responsibility and denies false assertions of action or inaction [24]. Additionally, specific types of attacks, such as listening and service spoofing, are linked to the breaches they can cause in the security requirements, emphasizing the importance of a comprehensive and layered approach to cybersecurity in energy management systems.

The architecture shown in Figure 3 illustrates the security requirements, hazards, and attacks in a microgrid system [24]. The security requirements are categorized into four primary domains: confidentiality, integrity, availability, and non-repudiation. Each criterion is complemented with a roster of specific hazards. An example of a situation where the principle of ‘confidentiality’ might be violated is via ‘unauthorized access to information’, which can be achieved using techniques like eavesdropping and interception. Instances of ‘unauthorized modification or theft of information’, such as masquerade and repudiation, weaken the idea of ‘integrity’. ‘Availability’ refers to the obstruction of allowed access or denial of service, which includes actions like service spoofing and resource depletion [24]. Finally, ‘non-repudiation’ ensures ‘accountability and denial of action’ to prevent denial of service attacks and disprove allegations of actions not carried out, as well as mitigate hazards such as repudiation and retrospective denial. Comprehending this comprehensive picture is crucial for developing robust security measures to defend microgrid systems from various cyber threats, ensuring a reliable and secure energy distribution network [24].

According to the IEC 62351 standard, the main security risks to microgrids can be divided into two categories: cyber and physical [24]. Cyber threats involve unauthorized access to communications, databases, and software, while physical threats include hardware, buildings, and people. It is important to note that security policies should be implemented at all levels to guarantee the safety, reliability, accessibility, and non-repudiation of the microgrid, as seen in Figure 3. In particular, the security of network connections is of utmost importance, particularly for IT networks, where confidentiality and integrity must be maintained, and industrial grid networks, where confidentiality, integrity, and availability are all essential [24].

### 2.2. Centralized vs. Distributed Microgrid Communication Networks

**Figure 4:**
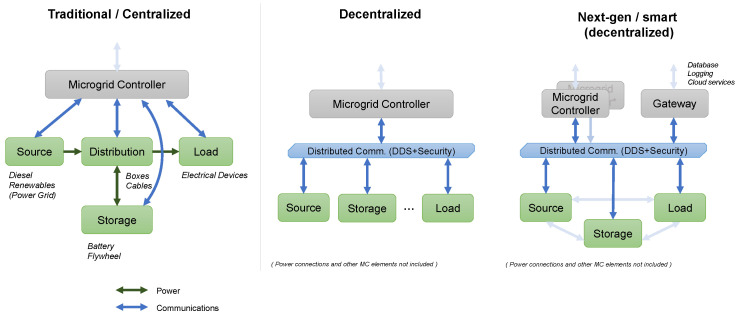
Network topology of microgrid data communication infrastructure.

A microgrid’s communication network may have either a centralized or a hierarchical structure, as illustrated in Figure 4. These electrical systems are flexible and resilient, and may be used either in conjunction with the primary power network or autonomously. They can range from small residential setups to larger groups of residences. Centralized and distributed approaches are often employed for network topology in microgrid management. All data in the microgrid network are managed by a centralized system, known as an energy management system. If there is a single issue in this system, it can have a major impact on the entire network. In a distributed system, each microgrid works independently, thus increasing its ability to withstand disruptions and ensuring consistent performance [25].

A centralized microgrid system utilizes a single controller to simplify and consolidate activities, thus decreasing operational conflicts and scheduling issues. On the other hand, decentralized systems allow for the autonomous functioning of multiple devices, leading to a considerable rise in redundancy rates. Microgrids employ a mix of wired and wireless connectivity methods. It is essential to precisely monitor and regulate parameters such as current, voltage, and power at each individual unit through suitable communication lines. This ensures the consolidation of authority and efficient supervision of microgrid components [26]. Effective communication is essential for effectively managing variations in power production and demand, as well as tackling critical distribution operating challenges such as voltage regulation and power flow control. For instance, solar photovoltaic farms may experience sudden changes in power generation due to shifting cloud cover, while wind farms may experience unexpected power loss. This infrastructure guarantees uninterrupted connection and the smooth exchange of information between all units. In a microgrid, a central controller makes it possible for devices in the central control topology (shown in Figure 4) to talk to each other at the same time. Regional central controllers collect information from multiple nodes and relay data towards the central controller. The process of choosing the communication technology or network architecture in a microgrid does not follow a predetermined methodology [27]. The secure and stable operation of the microgrid is heavily dependent on the use of effective communication technologies.

### 2.3. Cybersecurity Challenges in Microgrid Systems

Efficient microgrids, which combine physical and cyber systems, require dependable and efficient monitoring and administration. However, the integration of these components can create weaknesses in the system, necessitating a particular focus on these issues to ensure a successful deployment and operation of the microgrid. Smart microgrids are composed of complex arrangements, including distributed sensors, actuators, controllers, and power components, all of which require precise and prompt communication coordination. Smart microgrids must address challenges such as ensuring reliable connectivity, enhancing data security, and effectively managing large-scale data processing. This paper provides a thorough examination of existing standards and regulations related to microgrid cybersecurity. The IEC 62351 standard outlines key security risks in microgrids, such as protecting data confidentiality, preventing unauthorized alteration or theft of information, guaranteeing the availability of information and communication channels, and verifying the validity of communication [28].

Microgrids are exposed to a variety of attacks, including cyber-attacks that target communication networks, data storage, and software, as well as physical sabotage that can affect equipment, infrastructure, and personnel. Cybersecurity threats can also compromise the privacy, reliability, and accessibility of both IT and industrial automation systems. Fortunately, advances in network security have improved the security and operational efficiency of communication protocols in smart microgrid systems. This has enabled faster and more precise device interactions, as well as improved malfunction monitoring and troubleshooting.

Secure connection solutions are developed to meet certain requirements and objectives in smart microgrids. These microgrids usually have a three-tier structure, with an energy management system at the highest level, local controllers in the middle, and IoT devices such as smart meters at the lowest level. Each tier has its own computational and latency needs, which are addressed by various network nodes and standards. This article provides a comprehensive analysis of the communication needs, structure, protocols, and methods for ensuring secure connection in microgrids. It ends with a case study on the use of wide-area wireless technologies to improve the efficiency of microgrid services. Microgrids have the capability to function in either a linked or an independent manner, and may be adjusted in size to suit anything from individual residences to vast urban areas. Effective communication, either through physical connections or wireless technology, is essential for the immediate management and reliability of the microgrid. The communication infrastructure must provide uninterrupted connection, instantaneous performance, and security across all microgrid components.

### 2.4. Microgrid Network Topology Types

**Figure 5:**
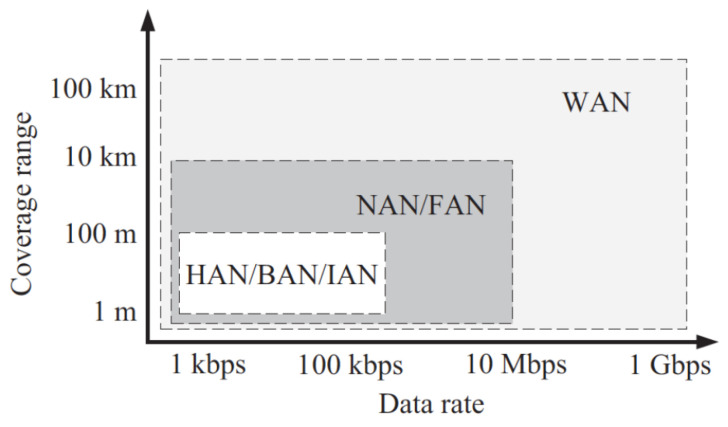
Subnet networks [29].

Microgrid systems use HANs, NANs, IANs, and BANs. Intelligent meters, generators, energy, and residential automation equipment constitute HANs. The more comprehensive IANs and BANs have extra automation instruments and sensors for development and commercial EMS and SCADA platforms. Several microgrid connectivity techniques differ in terms of service reach and transmission bandwidth, as seen in Figure 5 [29]. Deployment is possible, since these types of networks require minimal data transfer rates, energy usage, adaptability, and connectivity reliability. The quantity and kind of users might separate microgrids into customer-facing area networks, NANs, or FANs [29].

Table 1 presents a comprehensive overview of the communication technologies used in microgrid systems, along with their respective standards and applications. Technologies such as narrow band PLC, broadband PLC, DSL, and VDSL are listed with their corresponding standards, such as IEEE P1901.2 [30] and G3-PLC. Applications range from HAN to WAN. Advanced technologies, like WiMAX and cellular communications, with standards up to 5G, are also included. This research demonstrates that microgrids may provide effective connectivity capabilities for diverse networking capacities and data rates. HAN connectivity permits the creation of less extensive network connectivity. It provides access-level control of homes, enabling users to regulate energy use [29]. HAN is utilized for consumption management, heat operations, HVAC, intelligent meters, or home security system surveillance. These interactions are made feasible via equipment in buildings or neighboring sites that provide 100 kbps internet rates within a distance of 100 m and consume fewer watts of power. HAN uses Wi-Fi, Bluetooth, and ZigBee.

**Figure 6:**
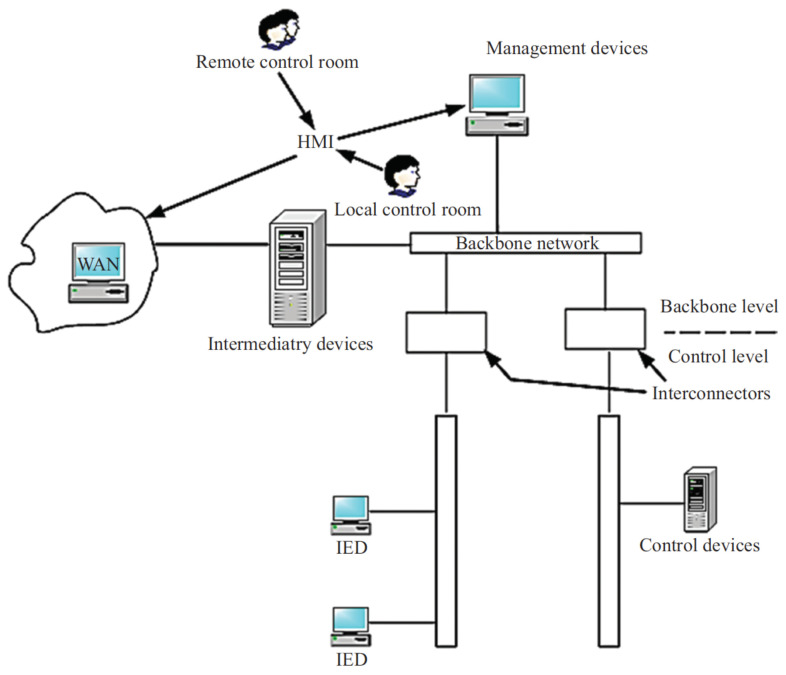
BAN network architecture [29].

The illustration in Figure 6 displays a two-level hierarchical system, referred to as a BAN, which is composed of a backbone and a control level with sensors, actuators, and controllers [29]. Internet connectivity is essential for BAN connectivity, allowing the sensors to collect data from the field at lower connection speeds. Interconnection devices (ICDs) enable the sensors to communicate with management devices at gigahertz speeds. The top tier of the BAN is made up of intelligent devices that monitor, log, document, query, and store process data values. A human–machine computing system located on the upper level produces charts and analytics reports for the management and customization of system assets. It has a user interface that can obtain data from the network and the environment. Additionally, operators can use the human–machine interface to query field equipment. Due to their location inside buildings, BANs use a low-power battery system that has little financial and environmental impact. Furthermore, BANs can communicate through ZigBee and Ethernet.

Near area networks (NANs) enable devices to communicate with each other more quickly due to their close proximity, allowing for faster data transmission rates of 10–100 Mbps within a 10 km radius. NANs are used at the distribution level in smart microgrids [29], and can be connected to devices via wired or wireless methods. They are also used by distribution network data collectors to communicate with intelligent meters. Better than hotspots, NANs allow for devices over a broader area. Further study is required to establish NAN information transfer throughput and rate. A local area network (LAN) is a collection of interconnected equipment, such as machines, in a specific region, such as a company, workplace, educational institutions, or set of homes [29]. A typical network has workstations, PCs, printers, scanners, and data storage devices. LAN devices can communicate quickly without leased lines. Local area networks employ Ethernet and Wi-Fi. LAN applications include an airport’s internet network, which lets passengers surf the web. LANs may be ring-based, star-based, mesh-based, or tree-based. LANs need routers, detection of network breaches, and the balancing of loads for privacy and security. LANs may regulate communication efficiency and information transfers.

**Figure 7:**
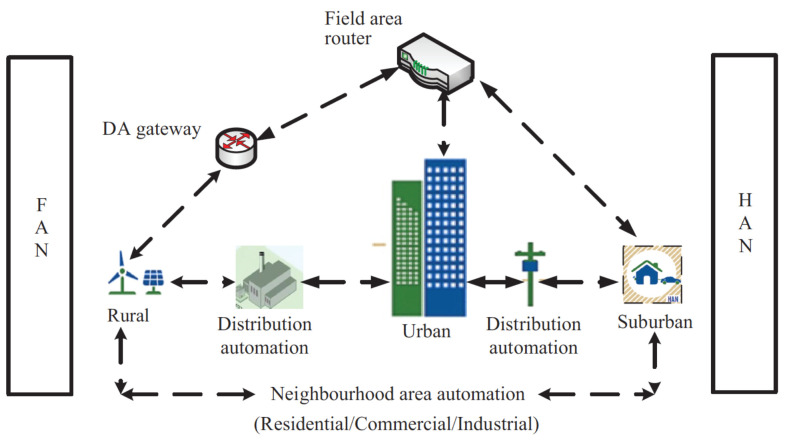
FAN network architecture [48].

Figure 7 illustrates a FAN architecture that enables a connection between a single endpoint and several points within a decentralized control system. This architectural style is gaining popularity at an escalating rate. The system relies on the NAN and HAN network designs. The FAN technology offers a wide range of uses, including energy production, intelligent metering, the administration of assets, or troubleshooting. As stated in reference [48], FAN is an economical solution that offers a superior degree of access to information and quality of service (QoS). Wide area networks (WANs) are telecommunications networks that cover a large geographic area. These networks provide more efficient control, protection, and monitoring over a larger area and are typically established using leased telecommunication lines. The communication speeds of WANs range from 1 Mbp to 1 Gbp [49]. WANs are the core layer of a communication system, connecting to all network nodes, including FANs and NANs, and typically have a 100 km radius. Wide area networks (WANs) can provide a reliable communication system by connecting multiple local area networks (LANs) with gateways at the end of the leased line. Two types of switching are employed in wide area networks (WANs): circuit switching and packet switching. Circuit switching creates a dedicated connection between two nodes, allowing for point-to-point communication until the call is terminated. Packet switching, on the other hand, sends data packets to each node and allows IEDs to receive them; however, this approach is more prone to errors, losses, and delays. Voice transmission is the primary application for circuit switching, while packet switching is used in networks.

### 2.5. Communication Infrastructures

The microgrid communication network can be either wired or wireless, depending on the device capabilities, the geographical region, and the available funds. Wired communication is the most straightforward option and can be achieved through power lines, twisted pair cables, and optical fibers. RF or cellular networks enable more complex wireless communication. Power line communication (PLC) technology uses power lines as signal carriers [29]. It was created in the early 1900s as a low-data-rate remote power network component control service. Since then, numerous frequency ranges and signal modulation methods have been used to reach data speeds from a few bits per second to 200 Mbps with a broad frequency range (3–20 MHz). PLC technology is susceptible to electromagnetic noise from electrical motors, radio signal interference, and power supply since power lines are not twisted and protected. Open circuits on the power line with switches and insulators can also cause disruptions of the connection. Physical grid architecture, impedance variations, and the reflection of the terminal point wave can weaken and distort signals, preventing transmission [29].

Twisted-pair wires made of copper have been widely employed in communication, from local area networks to telephone lines [41]. This cable transmits and receives electrical signals using one or multiple pairs of wires with plastic insulation. One of the wires is used to send the signal, while the other serves as a ground reference. Depending on the type of protection, twisted-pair communication cables can be unshielded, shielded, foiled, FTP, or S-FTP. The shield is composed of metal foil and braided mesh, which covers all conductor pairs or sets. This EMI shield helps to prevent noise and crosstalk from entering the communication channel. Despite its limited range and 1.54 MHz channel capacity, the twisted-pair cable is still an economical communication method. However, it has some drawbacks, such as lightning, water infiltration, power outages raising ground potential, cable failure, and difficulty in locating malfunctions.

In the 1960s, optical fiber replaced copper-wired connections in communication networks [41]. Common components of these systems include PON, WDM, SONET, and SDH, as noted in [41]. Fiber optic cables provide high data transfer rates (5, 10, 20, or 40 Gbps), immunity to RF and EMI, and the capacity to transmit data over long distances with fewer repeaters (100–1000 km) for electrical system automation. Optical fiber technology is beneficial for connecting electrical substation SG applications and communication networks. Despite its high installation cost, the technology’s high bandwidth capacity allows many users to share one communication channel as a backbone, making it more attractive. This makes optical fiber communication dependable and rapid [41].

Wireless communication technologies provide several benefits for microgrid operations in high-density areas by eliminating the need for intricate wiring infrastructure. Wireless solutions simplify operational management, allow greater flexibility in system design, and facilitate installation. A range of wireless standards that are appropriate for microgrid applications are listed in Table 1. These include IEEE 802.11 [42] for WLAN, which offers Wi-Fi connectivity for local area networks; IEEE 802.15 [44] for WPAN, which facilitates device-to-device communication via Bluetooth and ZigBee protocols [43]; and IEEE 802.16 [45] for WiMAX, which enables wide area coverage. Microgrids can benefit from cellular technologies that offer a wide range of reliable connectivity, ranging from 2G to 4G standards. However, each technology has its own difficulties, such as radio frequency interference, the need for a direct line of sight, environmental obstructions, and susceptibility to weather conditions. These elements must be taken into consideration when constructing and executing a dependable and effective wireless communication system for microgrid settings.

## 3. Micro-Grid Communication Protocols and Standards

**Figure 8:**
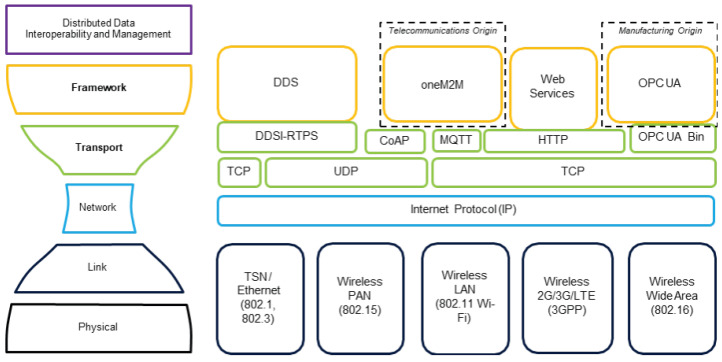
Connectivity standards [50].

IIoT standards are provided by the Industrial Internet Consortium (IIC). Volume G5 [50] explores the Internet 4.0 Internet Connectivity Stack paradigm, which unifies the three host levels into one framework layer, comparable to the Open Systems Interconnection (OSI) paradigm. Figure 8 compares OPC UA, DDS, oneM2M interface protocols, and Web Services. DDS is managed by the Object Management Group (OMG), which prioritizes quality of service (QoS) for smooth peer-to-peer communication [51]. Data are prioritized above semantic interoperability. DDS is planned for the OPC Unified Architecture (OPC UA) framework to provide publish/subscribe capability. OneM2M is a communication standard for mobile applications with intermittent connectivity that require low latency and minimal delay and establishes semantic interoperability. Web services enable human–computer interaction on the Internet over HTTP. OWL allows for semantic interoperability. Designed for industrial applications, the OPC UA is object-oriented and semantically interoperable, unlike DDS. Mobile devices in NDE applications may benefit from OneM2M’s limited connectivity and reduced latency and jitter. Operator interfaces may store and retrieve component information via web services, which are ideal for interaction between humans and computers. Since standard NDE equipment does not require quick and constant communication, we will not study DDS further. OPC UA is the preferred interface for NDE 4.0 in manufacturing due to its semantic interoperability and the status of the standard protocol [50].

### 3.1. The Role of IPv6 in Microgrid Cybersecurity

Integrating Internet Protocol version 6 (IPv6) into microgrid cybersecurity is a crucial development that guarantees secure and efficient communication [52]. IPv6 provides substantial enhancements compared to its predecessor, primarily in terms of authentication and encryption capabilities, due to its vast address space and advanced security features. Secure communication methods are crucial for maintaining operational integrity and safeguarding against cyber attacks in distributed microgrids. The implementation of IPv6 enhances the strength and scalability of network structures, allowing for improved management of Distributed Energy Resources (DERs) and more resilient communication networks. By utilizing the functionalities of IPv6, distributed microgrids can attain enhanced levels of security in their control and communication protocols, which is imperative in the progressively networked environment of contemporary power systems. IPv6 is utilized within the framework of Distributed Microgrid Cybersecure Control Communication Protocols and Standards, emphasizing its significance in strengthening microgrid networks against cyber vulnerabilities [52].

### 3.2. DDS—Data Distribution Service

The Object Management Group (OMG) has developed a middleware protocol, the data distribution service (DDS), to offer efficient data communication with minimal latency, maximum dependability, and the capability to manage large volumes of data. This system operates by enabling publishers to transmit data accompanied by a particular subject, allowing only subscribers seeking material related to that topic to access and read it. QoS requirements may be used to provide safe data sharing by including dependability, liveliness, authentication, access control, confidentiality, and integrity measures. The DDS system employs a decentralized peer-to-peer structure, and its security measures are detailed in a specification document (OMG, 2018) [51]. The DDS is a widely used technology that enables seamless, instantaneous, compatible, safe, and platform agnostic distribution of data across interconnected components. The DDS offers an application programming interface (API) for a data-centric, distributed, topic-based, real-time publish/subscribe paradigm. It is designed to provide high performance, support dynamic architectures, and enable real-time data transmission. Its focus on data makes it particularly suitable for the IIoT. Middleware facilitates the transmission of data across different applications and systems. Data centricity guarantees the inclusion of contextual information in all communications, enabling applications to comprehend the data. The essence of data centricity is in the fact that DDS has knowledge of the data it holds and exercises control over how those data are shared. Programmers using conventional message-centric middleware are required to develop code for transmitting messages. Programmers using data-centric middleware compose code that precisely defines the manner and timing of data sharing, thus facilitating the direct sharing of data values. Instead of handling the intricacy inside the application code, DDS provides a direct implementation of regulated, managed, and secure data exchange [51].

**Figure 9:**
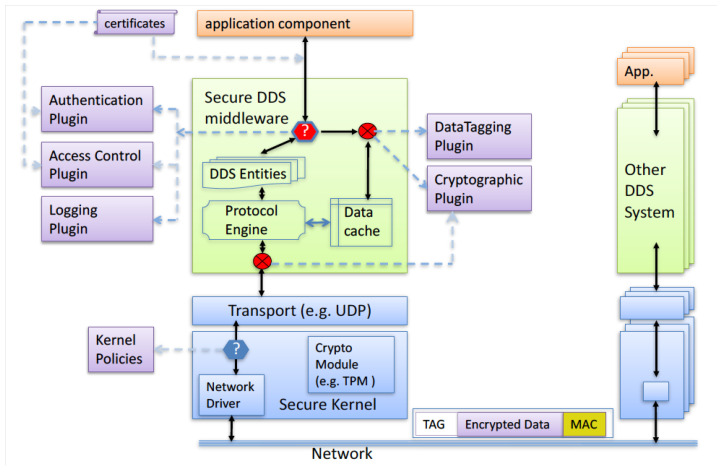
DDS security architecture [51].

DDS Middleware is the software layer between the OS and applications in a distributed system [51]. It facilitates seamless communication and data sharing among the system’s components. By abstracting the intricacies of inter-application and inter-system communication, it streamlines the process of developing distributed systems for software developers, enabling them to concentrate on the core functionality of their applications. As shown in Figure 9, implementing DDS security guarantees the essential levels of privacy, reliability, and accessibility that enable the deployment of the microgrid. The solution assigns a trust level to each device and implements topic access control inside the given DDS domain control. The security elements of DDS include authentication via the use of Public Key Infrastructure (PKI) and the Digital Signature Algorithm (DSA), together with Diffie–Hellman for authentication and key exchange. A commonly trusted certificate authority (CA) authenticates a permissions file, which serves as the foundation for access control. Clients have the capability to access and manipulate data subjects, as well as manage their involvement in systems. Cryptography is employed for secure key distribution, utilizing AES encryption and HMAC-SHA for message authentication and integrity. Data tagging is implemented with security metadata tags, which include classification level information. Security events are logged and securely distributed over DDS.

DDS is a conceptually organized publish/subscribe model that offers a continuous flow of information for distributing data. The system incorporates a range of quality of service (QoS) regulations to manage the fundamental, time-related, and geographical characteristics of data, such as resource utilization, real-time limitations, and priority. Moreover, DDS is compatible with several platforms and is backed by numerous programming languages, such as Java, Cpp, and Python, through an application programming interface (API). The OMG DDS architecture is engineered to exhibit scalability across a wide range of devices, from small to cloud-based systems, and to accommodate very large-scale deployments. It enables seamless communication among thousands or even millions of participants, facilitating the rapid transmission of data at exceptionally high speeds. Additionally, it effectively handles a vast number of data objects, while ensuring both high availability and robust security measures.

### 3.3. Industrial Data Spaces

**Figure 10:**
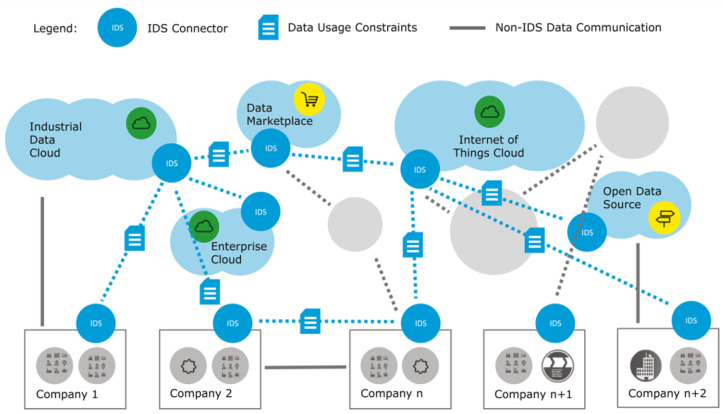
IDS ecosystem overview.

The International Data Spaces (IDS) initiative, led by the International Data Spaces Association (IDSA), is driving the transformation of the global digital economy [53]. IDS provides a secure and sovereign data-sharing infrastructure that allows all participants to benefit from the full value of their data assets. This infrastructure enables the development of new smart services and innovative business workflows that can operate across corporate and sectoral boundaries, ensuring that data usage remains under the control of data owners. As of 15 December 2023, considerable progress has been made to speed up the creation of data spaces, with ongoing improvements in data connector technologies being a major focus (see Figure 10).

**Figure 11:**
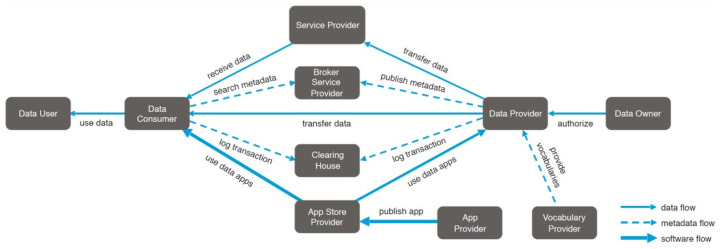
IDS data interaction [53].

The illustration in Figure 11 shows the key interactions that take place in the IDS concerning the flow of data. The exhibit showcases the responsibilities and interactions among the various entities, with the exception of the Certification Body and Evaluation Facilities, since they do not play an active part in the day-to-day operations of IDS. The Software provider is linked to all other positions via the ‘provides software’ relationship, whereas the Identity Provider is linked to all other roles through the ‘provides identity’ relationship [53]. This diagram illustrates the fundamental interactions among the roles in IDS. However, for the purpose of data transmission, further, more particular interactions are required.

The Identity Data Sharing (IDS) system empowers data owners with digital sovereignty, giving them control over ownership and data management, therefore facilitating smart services and creative business processes. The primary prerequisites for an Intrusion Detection System (IDS) are trustworthiness, security, data ownership, a network of interconnected data, standardized compatibility, value-enhancing applications, and data marketplaces. IDS is a widely accepted protocol for safe data transmission between companies that requires strict regulations on data management, ownership, and jurisdiction. The use of microgrid communications is a subject of ongoing study, especially in regards to outbound microgrid communications, where viable solutions are being explored.

### 3.4. Microgrid Specific Communication Standards

The IEC 61850 comprises a set of specifications developed by the technical panel associated with IEC 57, especially for systems that automate power substations [24]. The primary idea of IEC 61850 is the creation of information entities and services that are independent of any specific standards, allowing them to be easily modified to work with another standard, which meets the required requirements for information and services.

Common data classes (CDCs) are used to generate comprehensive, abstract data entities. The standard also offers instructions for aligning abstraction information and features with the Manufacturing Messaging Specification (MMS) standard, as well as aligning sampled measured values with the Ethernet data frame. These mappings provide communication between specific points or several points, allowing for both one-way and two-way transmission. The Substation Configuration Language (SCL), which is built on the Extensible Markup Language (XML), is employed to precisely depict the links between the automation system and the substation. IEC 61850 provides a comprehensive structure for organizing data that may be seamlessly used with any manufacturer’s equipment in the power system. This framework reduces the duration and exertion needed for device configuration. In order to do this, the SCL setup information is loaded to the device itself, allowing the IEC 61850 user application to remotely access the device’s physical properties.

The IEC 61850 protocol offers many advantages compared to proprietary protocols, such as compatibility with equipment from different manufacturers, reduced costs for installation, setup, and maintenance, improved scalability, and the potential for further developments in process automation. Additionally, IEC 61850 mandates a maximum latency of 4 ms, ensuring the quick transmission of overlapping tripping instructions with information sampling signals. This is necessary due to the time it takes for a protection message to travel from a specific logical node in the detection IED to the predefined distribution point at the physical network for the safety IED. Data packets can be sent either in the form of Multimedia Specification [54] or as sampled measurement values. This rapid transmission of information has a significant impact on the effectiveness and cost reduction for users in terms of operational tasks, such as updates on status, gathering information, time synchronization, or information transmission. The IED converts signals from power components, such as frequency, voltage, current, and watts, into a data representation that follows an object-based method. This facilitates operational convenience and enables improved control.

**Figure 12:**
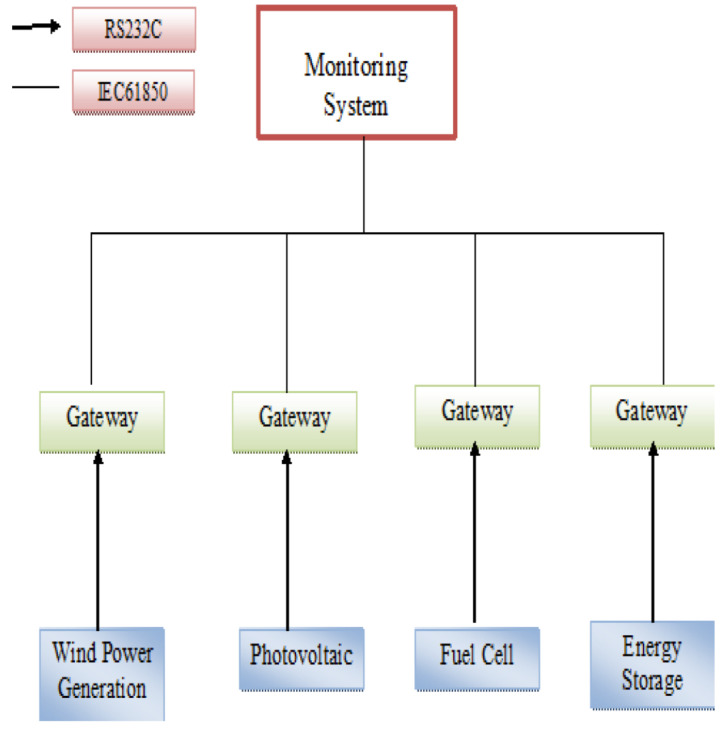
IEC 61850 based microgrid communications [38].

The figure shown in Figure 12 shows the many constituents of DER, including wind turbines, solar panels, fuel cells, and energy storage technologies. Each node is equipped with an individual controller that sends encoded measurement and status data, using a distinct protocol, to a gateway. The gateway changes the incoming values and status information into the IEC 61850 data model format and sends it to the microgrid monitoring system via a 100 Mbps Ethernet interface, taking advantage of the features provided by IEC 61850. The microgrid monitoring system operates as a compact Energy Management System (EMS), transmitting control orders to the DER controller via the gateway as necessary. The gateway serves two main purposes: it is the main hub for the Distributed Energy Resources Control Unit through a serial connection, and it functions as an IEC 61850 server for the microgrid monitoring system through an Ethernet link. Currently, 24 distinct IEC 61850 servers are being used to monitor various DER control units. The gateway collects data from a DER controller through a serial connection using a proprietary protocol that was developed as part of this research [39].

The IEC 61850 standard suite, created by the International Electrotechnical Commission (IEC), plays a crucial role in automating power systems and ensuring compatibility across devices in substations and microgrids [38]. The IEEE 2030 standard offers a comprehensive framework for achieving compatibility and seamless integration between energy technology and information technology within the entire power system, from power generation to consumption [55]. IEEE 1547 provides a collection of standards for connecting dispersed energy resources to electrical power networks. This ensures that microgrids may connect to and co-exist with the larger grid in a standardized and harmonized way [56]. The IEC 61968/61970 standards, called the Common Information Model (CIM), establish guidelines for data exchange between electrical distribution systems and distributed energy resource management systems. These specifications are essential for the smooth functioning of microgrid operations [57].

The IEC 62351 standard provides a comprehensive framework for security protocols in microgrid communication. It specifically focuses on ensuring that the security requirements of operational and information sharing protocols are met. The IEEE P2030.7 standard outlines the specific criteria for microgrid controllers, with a particular emphasis on the control elements of the microgrid operation, such as automation, stability, and energy management [58]. DNP3, or Distributed Network Protocol 3, is a collection of protocols used for communication inside process automation systems. It has gained popularity in microgrids due to its ability to provide secure and efficient communication. The comparison of the main aspects of several microgrid communication protocols is presented in Table 2.

Each standard fulfills a distinct function within the microgrid communications ecosystem, which includes many features such as interoperability, security, data transmission, and system integration. Comprehending these distinctions is essential for the efficient development and functioning of microgrid systems.

### 3.5. Microgrid Communication Protocols

The successful functioning of a microgrid is contingent upon the effective transmission of information, including the current state of the power system, its historical data, and its applications. The data are sourced from the communication system, emphasizing the crucial significance of communication protocols. The communication system enables the transfer of data across various components of the grid, including substation equipment DERs, and control centers, by adhering to a standardized set of rules governing data format and transmission. The power sector has developed many communication protocols to accommodate the diverse communication needs of different grid applications.

Modbus was originally developed to facilitate data interchange between Programmable Logic Controllers (PLCs) [59]. Since 2004, Modbus-IDA automation equipment suppliers and users have been responsible for the management and maintenance of this publicly accessible resource. The protocol operates at the application layer and employs an architecture based on client/server relationships, whereby the client device starts making an inquiry to the server in order to perform a specific action. Modbus utilizes a head multiplexer in a network using gateways to combine multiple transmission interactions, enabling the management of various system setups with one central device supervising multiple slave units [59].

**Figure 13:**
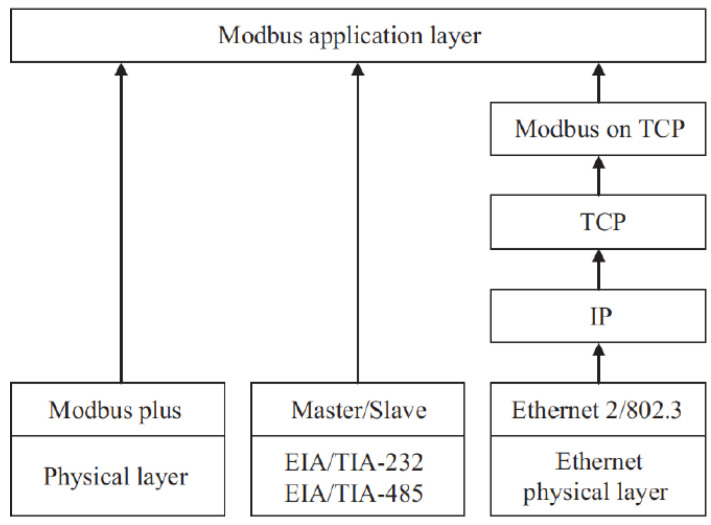
Modbus protocol layers [59].

The Modbus layer model is depicted in Figure 13. Modbus may be implemented on TCP/IP over Ethernet and other interfaces. This arrangement encapsulates data in TCP byte using Ethernet. The CSMA-CD multiple access carrier sense offers channel address and access control. Serial data transmission uses Modbus RTU, a 7-bit American standard encoding enabling information exchange. This kind of transmission needs double data traffic. This mode may be used in several forms of communication, including wired, fiber-optic, and radio channels.

In densely populated places, the presence of many interconnected devices in the wiring system might complicate the process of identifying problem locations. Wireless technology is favored for its installation, adaptability, and simplicity of operation, since it allows for the close proximity of equipment. The constraints associated with wireless technology include interference within the RF spectrum, line-of-sight limitations, the presence of physical barriers, and susceptibility to weather variations. The wireless technologies used for microgrids may be categorized as either short-range or long-range (cell), and are further explained below [60].

ZigBee technology enables wireless communication over small distances (100 m and 1600 m with ZigBee Pro), offering low-speed connectivity for personal networks [60]. The device works in the ISM band without requiring a license. The data rate varies depending on the frequency: 20 kbps for 868 MHz, 40 kbps for 915 MHz, and 250 kbps for 2.4 GHz. This technology is compatible with many network topologies and is applicable to automation systems in residential, commercial, and industrial settings. The cost of setting up and the energy used is minimal, and when combined with strong security protocols, it offers a dependable way to communicate. Nevertheless, the slow data transmission rate, restricted geographical reach, and potential disruption to alternative wireless solutions, such as Wi-Fi, hinder the widespread adoption of ZigBee technology to domestic use cases. Wireless local area network (WLAN) is a type of network communication that follows the IEEE 802.11 standards and is known for its fast speed [60]. It runs on 2.4 and 5 GHz ISM frequencies and transfers information at 2–600 Mbps. Wi-Fi is fast, safe, and reliable. WLAN technology is typically used for local networks in residential and business settings because of its restricted service area, high installation costs, and high energy consumption.

A wireless mesh network is a strong, cost-effective, and extensible network that connects to routers and mesh clients. Each point of the link acts as a repeater in this dynamic routing network, speeding up data packet delivery between nodes [60]. If a node fails, the connected topology allows the surviving nodes to communicate. Using IEEE 802.11 standards [42], wireless mesh networks cover large areas using radiowave reflection technologies. Home automation and AMR systems employ wireless mesh technology due to its wide coverage, robustness, and self-repair. Low-speed data transfers and wireless network interference are other factors that limit technology. Wireless network technology (Z-wave) is cheap, low-energy, and short-range. The system uses 900 MHz ISM frequencies and can carry data at up to 40 kbps across 30 m [42,60]. This technology automates residential and commercial lights. This low-power device is suitable for microgrid applications.

## 4. Utilization of DDS in Various Industries

**Figure 14:**
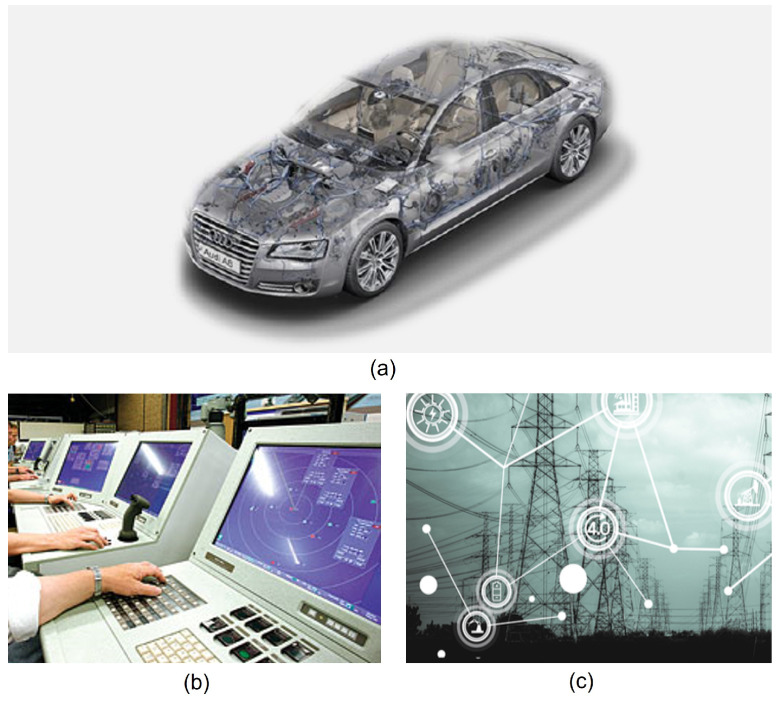
Deployment of DDS across diverse sectors including (**a**) automotive production [61], (**b**) SCADA reliant industries [62], and (**c**) electrical utilities [63].

DDS has become an essential standard for enabling high-performance real-time data transmission in a variety of business sectors. This technology is seen as a key element of modern infrastructure due to its ability to maintain complex, distributed systems that have stringent requirements for reliability and scalability. This section examines multiple case studies that illustrate the versatility and importance of DDS implementation in different operational contexts (Figure 14).

- In the automotive industry, especially at Volkswagen [61], there is a need to make the flow of data between high-end sensors and various subsystems more efficient while integrating with existing software ecosystems. Volkswagen chose RTI Connext DDS to accomplish this, using it as a middleware to create modularity in communications. The main benefit of DDS in this context is its capacity to separate the application layer from the communication infrastructure, allowing for easy relocation of processes across the network with automatic routing and discovery. This eliminates the need to modify IP configurations or network interfaces. The quality of service (QoS) features of DDS are essential in meeting the unique data delivery requirements of each module, managing discrepancies between publishers and subscribers without any difficulty.
- Siemens Energy [63] incorporated RTI’s messaging software into their SCADA systems to manage wind power generation, taking advantage of its scalability and dependability. This software provides a range of network services that span LAN, broadband, and satellite communications, allowing for a smooth integration of wind turbines with existing IT infrastructure for optimal performance. Not only does RTI’s software support real-time messaging and quality control for managing hundreds of wind turbines, but it also integrates with enterprise systems for remote monitoring and diagnostics.
- LocalGrid Technologies utilized DDS to bolster the security and effectiveness of SCADA-based communications across distributed microgrid nodes [62]. This technology guarantees that the data traffic is secure and that the network bandwidth is used optimally as the microgrid grows. Working with RTI and other partners, they are striving to develop the next generation of microgrid solutions.
- The Netherlands Railway System, Prorail, has implemented DDS to meet the rigorous requirements of its expansive transportation management systems. By combining telemetry with sensor data, DDS helps to ensure the real-time coordination necessary for the effective management of railways, trucks, and fleet operations. It provides a dependable data sharing platform that Prorail relies on to manage the crowded Dutch rail network [64].

## 5. A DDS Proof-of-Concept Implementation for Microgrid

### 5.1. Overview

By 2050, the Dutch government must reduce CO_2_ emissions by 80 to 95%. Energy from an exhausting source must be replaced by a sustainable source, such as wind energy and solar photovoltaics [65,66,67]. This will cause new problems: it will lead to irregular energy supplies. The challenge facing microgrids is the absorption of fluctuations in electricity frequency and local demand. Microgrids have the disadvantage of uncertain energy inputs and outputs. It is possible to increase electricity on windy days, or many homes will suddenly begin to use more electricity. Therefore, QuinteQ Energy BV is focused on developing flywheel technology to enable microgrid stabilization and flexibilization, working in partnership with TS, Twent University, and the Applied Science University of Sicily to find and validate solutions in real-world test scenarios [68]. The flywheel is a device designed to efficiently store rotating energy. It can store power by flowing power to the flywheel, which accelerates the rotating disc. The motor can also act as a generator when the current is reversed, slowing the disk and producing power. The project is developing control systems and communication protocols, control and control of flight wheel status, and other microgrid systems such as solar panels and batteries. The main task of the QuinteQ project is to make the flywheel capable of maintaining the appropriate state of the microgrid using flexible energy sources. That means that the flywheel can stabilize the microgrid at any time. It is important to stabilize the microgrid at any time, as sudden changes in electricity generation and consumption are always unpredictable and are not known in advance. This section focuses on studying DDS, a DDS-based communication protocol for implementing secure cyber communications in microgrids. After initial research, a framework will be selected and prototypes will be made with this framework. These prototypes are a small demonstration of exploring and verifying the cybersecurity aspect of this type of application.

### 5.2. System Requirements for Cybersecure Communication Middle-Ware

To ensure the efficient operation of microgrids, the chosen communication middleware must be designed to minimize delays, enabling rapid control actions that are essential for system stability. It should provide tailored service options to meet the specific needs of various types of data and control processes. In the context of microgrids, the middleware must be adjustable dynamically, including the capacity to detect new devices automatically and reconfigure in emergency scenarios without having to review protocols. Data-centric solutions, such as DDS, are preferred due to their smooth processing, peer-to-peer capabilities, and adjustable service configuration quality, which support scalable, secure, and dependable communication frameworks for smart networks.

Prerequisites for secure usability protocols and justification for adopting DDS:

- Enhanced security mechanisms incorporate layered defenses, integrated protective measures, vigilant threat detection, and operator-authenticated controls.
- Facilitation of simple operating procedures and maintenance.
- Enabling dynamic system reconfiguration capabilities.
- Commitment to open communication standards.
- Provision for real-time operational competence.
- Scalability and demonstrable dependability in field applications. The item conforms to the OMG DDS standards.
- Real-time operability, centered on data processing.
- Compatibility with conventional Ethernet frameworks.
- Modular development with decoupled components promotes ease of integration and adaptability.
- It is recognized for its application in critical sectors and supported by an open licensing model.
- Strong technological infrastructure with extensive QoS options, a secure and interoperable protocol, and a machine-readable Interface Definition Language (IDL).
- Sustained by a robust ecosystem that fosters steady governance, diverse commercial implementations, constant innovation, and wide utilization by the industry.

In order to guarantee secure usability protocols, the Distributed Data Security (DDS) system should be adopted. DDS provides a secure environment for data storage and communication and is designed to prevent unauthorized access. It also offers a secure authentication process, which is essential for secure usability protocols. Furthermore, DDS has a variety of features that make it a great option for secure usability protocols, such as encryption, data integrity, and access control. Consequently, it is sensible for organizations to adopt DDS to ensure secure usability protocols.

### 5.3. DDS-Based Communication and Modular Control Architecture

**Figure 15:**
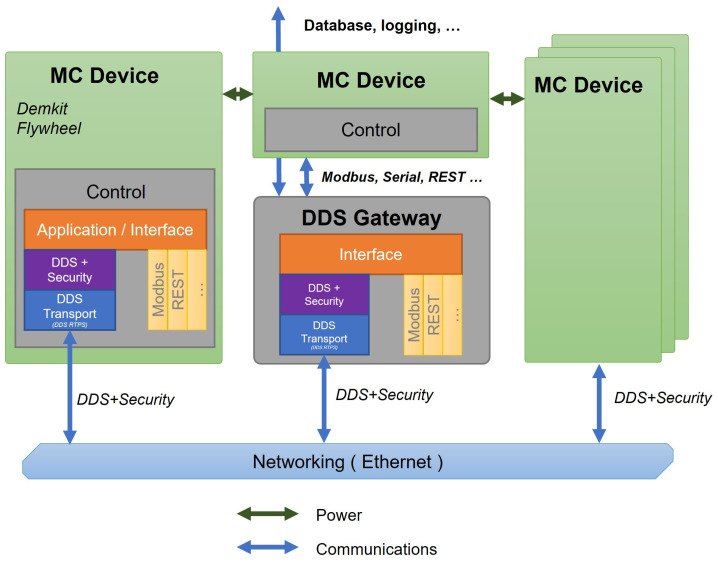
Implementation of DDS data collection and controller. MC stands for microgrid controller.

The diagram shown in Figure 15 showcases the constituent elements of the DDS framework, with particular emphasis on the incorporation of the DDS middleware. The text delineates the structure of the proposed DDS, together with the data gathering and control mechanisms. The communication framework leverages the functionalities of DDS, integrating strong security mechanisms and functions across networking interfaces, such as Ethernet connections. The gateway has a dual function: it acts as the central hub for the control unit of distributed energy resources and as a server for the microgrid monitoring system. The gateway obtains data from the controller using a connection link protocol and transmits them to the microgrid’s monitoring system utilizing DDS technology [39].

Multiple microgrid controller (MC) devices are connected by a DDS gateway, as seen in Figure 15. Every MC device is equipped with its own control mechanisms and connects to the DDS Gateway via an application interface, ensuring safe communication using DDS Security protocols. The DDS Gateway serves as the central component of the system, allowing the movement of data, ensuring security, and connecting with other network protocols such as Modbus, Serial, REST, and others. This gateway serves as the central point for data exchange, taking inputs from MC devices and securely sending them over the network, which is mostly based on Ethernet technology. The graphic highlights the bidirectional flow of communication, where power and data are exchanged between the MC devices and the DDS gateway, demonstrating a resilient and protected network for microgrid management.

### 5.4. Prototyping and Implementation

**Figure 16:**
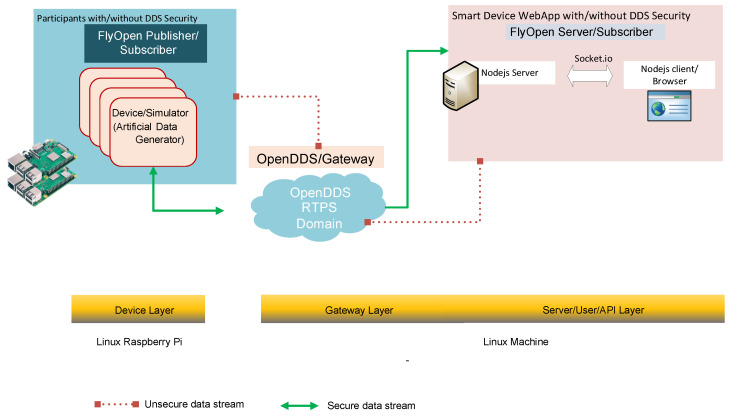
DDS standard implementation.

**Figure 17:**
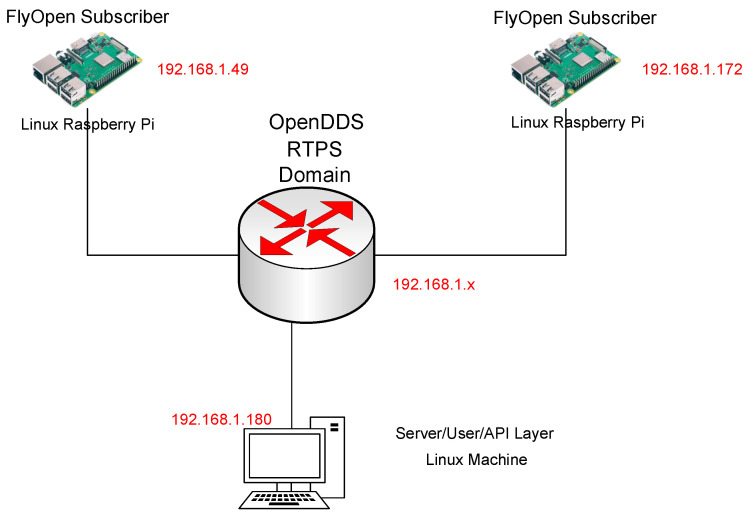
DDS PoC hardware setup.

Figure 16 and Figure 17 show the proof of concept implemented for DDS design demonstration, the test environment has three devices: smart subscriber, publisher, and local DDS Gateway. The publisher and subscriber are set up with in the same local subnet. They are configured to be in the same DDS DDS domain ID with the common topic identifier. A local router acts as a DDS gateway. Publisher is a simple smart device emulator that generates power data in [Kw] in a random manner. The publisher could emulate 10 different smart dice that were being sent simultaneously. The smart device server is a NodeJS DDS server that utilizes the DDS NodeJS module to receive and act as a DDS subscriber. The NodeJS server is able to collect to any HTTPS based web-socket requests to display the data that are received.

### 5.5. Configuration and Methodology of DDS Testing

The DDS performance assessment framework makes use of configuration files to determine testing behavior and network topology. These files are divided into two categories: transport configuration files and test configuration files. The transport configuration files provide individual keys for each transport instance and their parameters, while the test configuration files define the testing environment and network settings. Generally, a single transport configuration file is used to control each test, which specifies the parameters. Processes then select the relevant configured transport instances they require.

### 5.6. Assessment of Network Throughput

**Figure 18:**
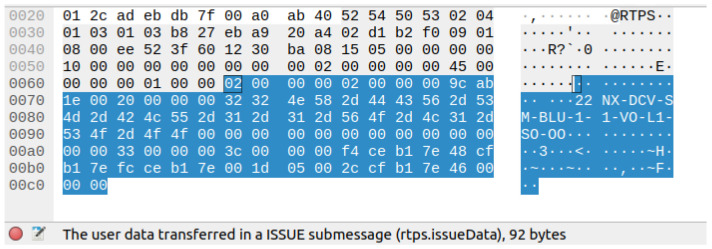
User data in the received DDS protocol 92 bytes, Wireshark.

**Figure 19:**
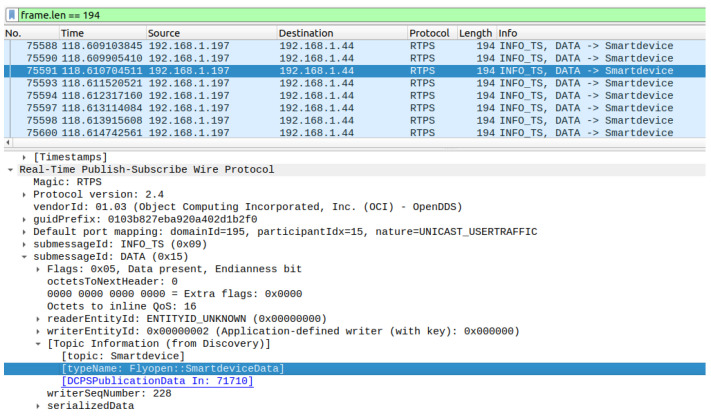
Received DDS protocol frames, wireshark filter (frame.len==194).

We conducted an analysis to assess the occurrence of communication packet loss, taking into account the data size and rate requirements of the user’s application. We tested the effectiveness of the DDS communication network using DDS middleware by performing experiments to measure network performance and identify instances of packet loss. We evaluated the performance of the Ethernet protocol by sending repeated sets of DDS data frames and measuring the amount of packet loss from the publisher to the subscriber. We replicated the process for both unicast and multicast configurations to determine the acceptable level of frame loss that would not impede the application’s performance. Figure 18 and Figure 19 illustrate the results, showing the length of the test in relation to the occurrence of packet loss, as recorded by Wireshark. The tests were conducted on time-shared servers, suggesting that deploying on hosts with real-time operating systems could further improve performance metrics.

**Figure 20:**
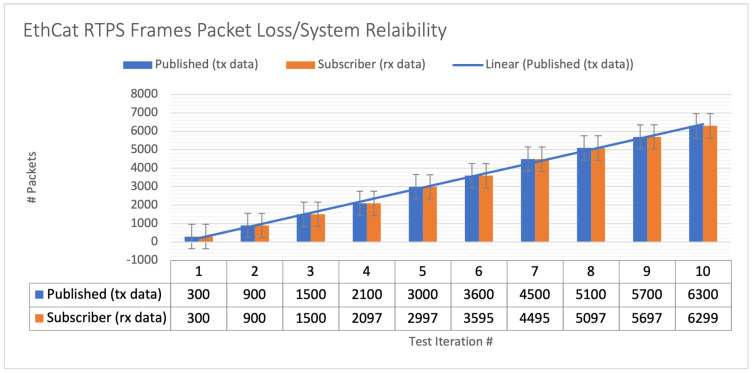
Performance analysis for DDS and best effort QoS.

**Figure 21:**
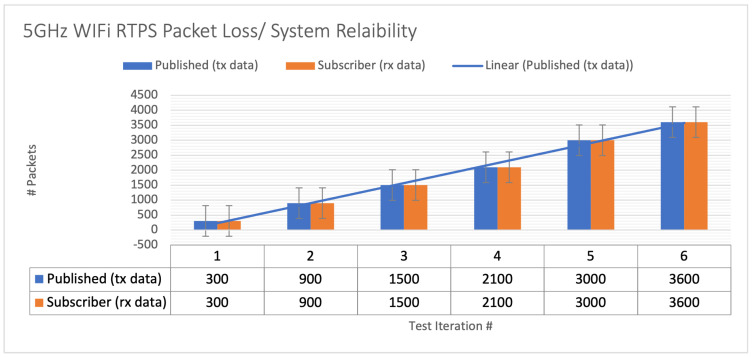
The 5 GHz WiFi DDS packet loss/system reliability performance analysis for DDS with best effort QoS setting.

The hardware setup for the DDS proof of concept (PoC) is demonstrated in Figure 17, while a Wireshark snapshot of user data sent across the DDS protocol is displayed in Figure 18. Figure 19 shows the DDS protocol frames that were received and filtered based on their frame length in Wireshark. The performance study, depicted in Figure 20, investigates the relationship between packet loss and test iterations for DDS while operating under a best effort quality of service (QoS) configuration. Additionally, Figure 21 provides an analysis of the occurrence of lost data packets and the dependability of the DDS 5 GHz Wi-Fi DDS connection. It displays the precise technical characteristics of the wireless interface used by both the subscriber and publisher. The observed correlation between the published and received data in DDS transport indicates a low packet loss rate, which is likely due to the lack of network congestion in the local testing environment.

**Figure 22:**
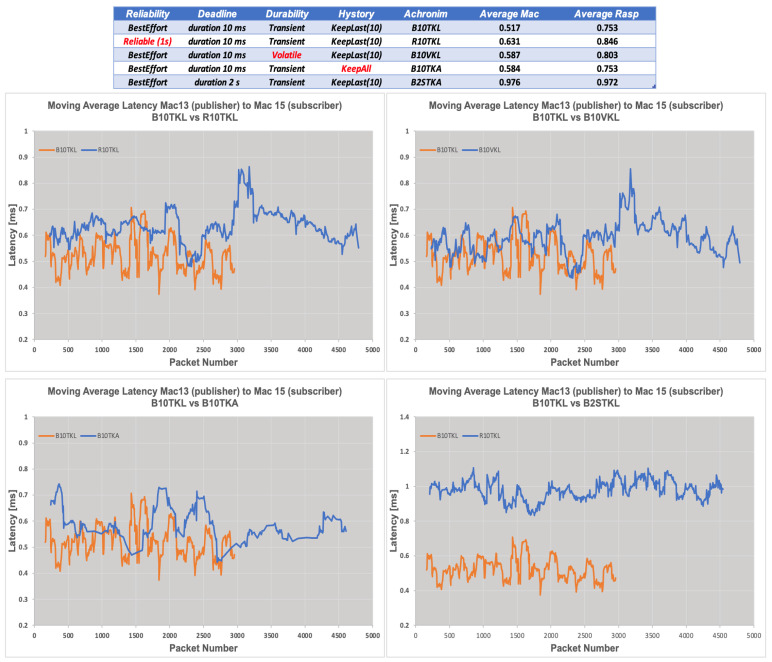
Comparison of moving average latency trends for different QoS policies in a publisher/subscriber model.

**Figure 23:**
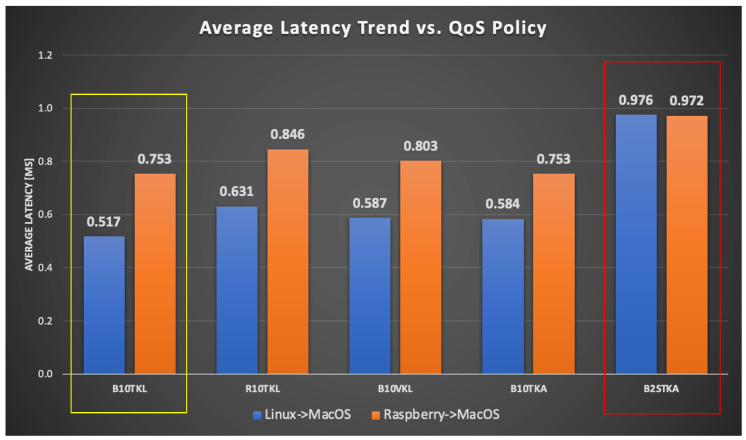
Average latency trend comparison against QoS policy between Linux and MacOS, and Raspberry Pi and MacOS systems. The yellow box highlights the best performance on Linux compared to MacOS, while the red box indicates the closest performance between Raspberry Pi and MacOS.

The graphs in Figure 22 and Figure 23 provide a comprehensive analysis of the moving average latency, considering different quality of service (QoS) policies, on two machines named Mac13 (publisher) and Mac15 (subscriber). The data illustrate the variations in latency trends across a range of packet quantities, demonstrating the effects of various quality of service (QoS) policies such as B10TKL and R10TKL. Latency, measured in milliseconds (ms), is a measure of the short-term performance of the communication system. The bar chart in Figure 23 displays the average latency values, providing a distinct visual representation of the contrasting performance across different combinations of operating systems across various QoS scenarios, including B10TKL, R10TKL, and B2STKA. The results demonstrate the latency that can be expected when implementing these systems with specific QoS criteria. The findings demonstrate that QoS policies have a significant impact on the latency experienced by the systems. For instance, B10TKL exhibits varying latencies when used with Linux and MacOS compared to Raspberry Pi and MacOS, as depicted in Figure 23. These insights are essential for improving real-time communication protocols in microgrid contexts, where rapid data transfer is essential.

## 6. Discussion

This article offers a thorough examination of the prerequisites, system architecture, and techniques for ensuring secure communication in microgrids. This publication addresses the latest developments and unsolved issues with communication protocols and standards in smart microgrids. A innovative, decentralized, and secure communication protocol, using the data distribution service (DDS), was created and assessed, including a case study and a proof-of-concept demonstration. The analysis emphasizes certain significant obstacles and unresolved research issues:

- Integrating security into a system can be accomplished through the use of the data distribution service (DDS). This security paradigm provides clear and compatible security rules at the middleware level, allowing for the use of smart devices while still maintaining flexibility, scalability, performance, and quality of service (QoS). This approach simplifies the process of creating secure system designs by allowing for the assignment of specific rights to DDS domains, topics, or data object instances inside a topic. The ‘partition’ quality of service (QoS) feature offered by DDS allows for the creation of separate subdomains, thus increasing the grid network’s resistance to attacks. This security strategy incorporates network segmentation and enhanced packet inspection to actively monitor the network in real-time and quickly deploy security applications to combat malware and DDoS attacks.
- Maximizing Middleware Utilization: Using a single middleware that can manage multiple communication patterns is an economical way to build and maintain large, distributed systems. The message-oriented publish/subscribe nature of DDS offers a broad range of communication patterns, making system development more flexible and allowing for efficient traffic classification and prioritization.
- Smart Device Design: The incorporation of low-capacity devices, such as wireless sensor network smart meters, into the metering system of the microgrid should be taken into account when designing smart devices.
- Data Management: The challenge of efficiently managing the transmission of large amounts of data from intelligent devices, particularly embedded sensors, is considerable. DDS offers a ‘multichannel’ approach to maximizing network traffic by splitting the data stream into multiple channels. Nevertheless, careful examination is required to facilitate rapid configuration modifications.
- Service Discovery: DDS discovery enables the linking of multiple intelligent devices by maintaining a comprehensive list of local items for network surveillance. This capability is critical for enhancing the compatibility of protocols and allowing for successful network management.
- Scalable Data Handling: In order to prevent overcrowding in systems with a large number of gateways, DDS offers support for data filtering and merging techniques. These approaches reduce unnecessary network activity and optimize the utilization of available bandwidth, allowing for the selective processing and transmission of data.
- Empirical testing: It is essential to carry out tests on actual devices to check if the middleware architecture is compatible with the desired environments.

## 7. Conclusions

This study presents a thorough examination of secure communication in microgrids, with a specific emphasis on the data distribution service (DDS). The text discusses important obstacles and outlines potential areas for future investigation, such as improving the security of DDS-based systems at the middleware level, optimizing the efficiency of middleware in distributed systems, and integrating low-capacity smart devices to enhance data management efficiency. The study highlights the significance of implementing scalable data management, enhancing network monitoring, and conducting thorough empirical testing to verify middleware architectures. The importance of implementing sophisticated communication frameworks in microgrids, namely to guarantee cyber-physical security and optimize real-time data management, is emphasized. It is essential to include advanced communication protocols and robust cybersecurity safeguards into future microgrid systems as the energy sector transitions towards sustainable sources. This evaluation establishes the foundation for future investigations in the creation of advanced, highly secure middleware solutions, with the goal of enhancing the durability and sustainability of microgrid systems.

## Figures and Tables

**Table 1:** Compilation of communication modalities in microgrid systems.

Modality of Communication	Standards	Applications
Narrowband PLC [29]	IEEE P1901.2 [30], G3-PLC [31]	HAN/NAN/WAN
Wideband PLC [32]	IEEE 1901 [33], Home Plug 1.0 [34,35]	HAN/BAN/NAN
DSL [36]	ADSL [37]	NAN/FAN
VDSL [37]	VDSL [37]	NAN-FAN
HDSL [38]	HDSL [38]	NAN/FAN
Ethernet [39]	IEC 61850 [28]	HAN/BAN/NAN, SAS [40]
Optical fiber [41]	PON [41], SONET/SDH [41]	WAN
WLAN [42]	IEEE802.11 [42]	Local Networks [43]
WPAN [44]	IEEE 802.15 [44]	Bluetooth, ZigBee [45], Home Networks
Cellular [46]	Generations 2 to 5 [46]	V2G, WAN/LTE-A [47]

**Table 2:** Comparison of microgrid communication standards.

Standard	Scope	Key Features	Application Area
IEC 61850 [28]	Substation automation	Interoperability, real-time data exchange, object-oriented approach	Substations, microgrids
IEEE 2030 [55]	Smart grid interoperability	Integration of energy and IT, guidelines for system integration	Smart grids, microgrids
IEEE 1547 [56]	Interconnection of distributed resources	Standards for connecting distributed resources to the grid	Distributed generation, microgrids
IEC 61968/61970 [57]	Data exchange in electrical distribution	Standardized data exchange model, integration of distributed resources	Distribution systems, microgrids
IEC 62351 [24]	Security for power system operations	Security protocols, authentication, encryption	Power systems, microgrids
IEEE P2030.7 [58]	Microgrid controllers	Standards for control, automation, and energy management	Microgrid control systems

## Abbreviations

The following abbreviations are used in this manuscript:

EMS	Energy Management Systems
RTU	Remote Terminal Unit
PLC	Power Line Communication
LAN	Local Area Network
IIC	Industrial Internet Consortium
DDS	Data Distribution Service
QoS	Quality of Service
OMG	Object Management Group
IDS	International Data Spaces
LAN	Local Area Network
WAN	Wide Area Network
EMI	Electromagnetic Interference
RF	Radio Frequency
PLC	Power Line Communication
API	Application Programming Interface
AMR	Automatic Meter Reading
MMS	Manufacturing Messaging Specification
SCL	Substation Configuration Language
IED	Intelligent Electronic Device
DER	Distributed Energy Resource
